# Acute plant-derived C18 fatty acid exposure was associated with changes in muscle 5-LOX protein abundance and volatile profiles in triploid rainbow trout (*Oncorhynchus mykiss*)

**DOI:** 10.1016/j.fochms.2026.100426

**Published:** 2026-06-01

**Authors:** Yongbang Si, Yongna Song, Dong Huang, Yifei Chen, Jun Sun, Zezhong Wu, Rui Ma, Guoliang Sun, Yuqiong Meng

**Affiliations:** a Cold-Water Fish Research Center, State Key Laboratory of Plateau Ecology and Agriculture, Qinghai University, Xining 810016, PR China; b Key Laboratory of Plateau Cold-water Fish Culture and Eco-environmental Conservation (Co-construction by Ministry and Province), Ministry of Agriculture and Rural Affairs, Qinghai University, Xining 810016, PR China; c College of Eco-Environmental Engineering, Qinghai University, Xining 810016, PR China

**Keywords:** Fish oil replacement, Lipid oxidation, Volatile organic compounds, Odor activity value, Sensory quality

## Abstract

Driven by sustainability, plant oils are increasingly used to replace fish oil in aquafeeds, thereby altering muscle fatty acid composition and potentially affecting volatile profiles. Lipoxygenases (LOXs) participate in fatty-acid bioconversion, but the role of 5-lipoxygenase (5-LOX) in non-immune fish muscle remains unclear. We hypothesized that acute intragastric exposure to plant-derived C18 fatty acids would be associated with changes in muscle 5-LOX protein abundance and volatile profiles in triploid rainbow trout.

Using an acute oral gavage model, trout received oleic, linoleic, or α-linolenic acid, alone or with the selective 5-LOX inhibitor zileuton. Muscle samples were analyzed for 5-LOX mRNA expression, detectable protein abundance, and volatile profiles.

Detectable 5-LOX protein abundance was low under basal conditions and changed after fatty-acid administration, with linoleic acid showing the clearest increase. Several major volatiles also changed across treatments. Under acute fatty-acid exposure, zileuton reduced hexanal concentration by 85.7% (221.2 to 31.6 ng/g) and decreased the nonanal odor activity value by 34.4%.

These results indicate that acute plant-derived C18 fatty acid exposure was associated with changes in detectable muscle 5-LOX protein abundance and volatile profiles in triploid rainbow trout. The inhibitor-associated volatile changes further support the possibility that multiple oxidative processes may be involved under these acute conditions.

## Highlights

• Muscle 5-LOX protein abundance was low under basal conditions and was altered following acute plant-derived C18 fatty acid exposure.
• Acute intragastric administration of linoleic and α-linolenic acids was associated with changes in muscle 5-LOX protein abundance.
• During acute intragastric fatty acid exposure, pharmacological blockade with zileuton altered the abundance of several major volatiles.

## 1 Introduction

The aquaculture industry is under increasing pressure to reduce its dependence on fish oil because of rising costs and limited global supply. As a result, terrestrial plant oils have been widely used as alternative lipid sources in aquafeeds (Gu et al., 2025). This nutritional shift alters the fatty acid composition of fish muscle, typically increasing the proportions of C18 polyunsaturated fatty acids (PUFAs), such as linoleic acid (LA, 18:2n-6) and α-linolenic acid (LNA, 18:3n-3), while reducing the levels of long-chain highly unsaturated fatty acids (LC-HUFAs), including EPA and DHA (Torstensen et al., 2005). Because muscle lipid composition is closely related to fillet quality, these changes may also affect the organoleptic properties of fish, especially aroma (Frank et al., 2009; Hashimoto et al., 2024; Selli & Cayhan, 2009; Xu et al., 2022). This question is relevant to triploid rainbow trout (*Oncorhynchus mykiss*), a commercially important species valued for rapid growth, sterility, and desirable flesh quality (Ma et al., 2019).

The aroma of fresh fish is largely influenced by volatile organic compounds (VOCs), including aldehydes, alcohols, and ketones generated during fatty acid oxidation and degradation (Ye et al., 2024). Due to their multiple double bonds, PUFAs are particularly susceptible to oxidation, and different fatty acid precursors can give rise to different volatile patterns. For example, n-3 PUFAs such as LNA have been linked to compounds such as (*E,E*)-2,4-heptadienal, whereas n-6 PUFAs such as LA are commonly associated with hexanal and 2-octenal (Yang et al., 2017; Yao et al., 2024). Therefore, replacing fish oil with plant oils may alter the balance of volatile compounds in farmed fish, partly through increased oxidation of plant-derived C18 fatty acids (Turchini et al., 2007).

In fresh muscle, volatile formation can proceed through both non-enzymatic and enzyme-mediated pathways. Among the enzyme systems involved, lipoxygenases (LOXs) are of interest because they catalyze the oxygenation of PUFAs containing a *cis,cis*-1,4-pentadiene structure to form hydroperoxides, which can serve as precursors of volatile compounds (Yang, Liu, et al., 2025; Yang, Sang, et al., 2025). In aquatic species, LOX-related activity has mainly been discussed in relation to the 5-, 12-, and 15-LOX isoforms. Although 12-LOX and 15-LOX have been associated with lipid oxidation in different biological systems, the specific contribution of individual LOX isoforms to volatile formation in edible fish muscle remains unclear, especially under in vivo conditions.

Among these isoforms, 5-lipoxygenase (5-LOX) remains insufficiently characterized in the context of volatile formation in edible muscle. Although its role in leukotriene biosynthesis in immune tissues has been well documented (RÅDmark, 2000), its possible contribution to fatty-acid-derived volatile changes in fish muscle has not been clearly defined. For this reason, 5-LOX was examined in the present study as a candidate enzyme potentially associated with volatile changes under acute fatty acid exposure.

We therefore asked whether acute exposure to individual plant-derived fatty acids would be accompanied by changes in detectable muscle 5-LOX protein abundance and shifts in volatile profiles in triploid rainbow trout. To address this question, we used a controlled in vivo oral gavage model with OA, LA, and LNA, and included zileuton as an exploratory pharmacological tool in the experimental design. Using this short-term exposure model, the present study aimed to provide preliminary evidence on whether plant-derived C18 fatty acids are associated with changes in detectable muscle 5-LOX protein abundance and volatile profiles in triploid rainbow trout. These findings may help inform future studies on short-term biochemical and volatile responses to plant-derived fatty acid exposure in fish muscle.

## 2 Materials and methods

This study was conducted in strict accordance with the standard operational procedures stipulated in the guidelines for the use of experimental animals at Qinghai University. The research protocol was reviewed and approved by the Ethics Committee of Qinghai University. Furthermore, all in vivo experimental designs, executions, and reporting strictly comply with the ARRIVE 2.0 (Animal Research: Reporting of In Vivo Experiments) guidelines.

All female triploid rainbow trout used in this study were obtained from a local fishery in Qinghai, China. Two distinct size classes of triploid rainbow trout were utilized in this study to address different experimental objectives. For the baseline physiological tissue distribution profiling, market-size sub-adults (average weight ∼ 3.0 kg) were sampled to reflect commercial production endpoints. Conversely, for the acute in vivo treatment trials (fatty acid gavage and pharmacological inhibition), a standardized juvenile model was employed to ensure precise dosage control under controlled experimental conditions. Specifically, a total of 135 juvenile fish with an initial body weight of approximately 50 g were reared in the Key Laboratory of Plateau Cold-water Fish Aquaculture and Ecological Environment Protection, Ministry of Agriculture and Rural Affairs, Qinghai University. Prior to the experiment, fish were acclimated for one month and fed a commercial diet twice daily at 11:00 and 18:00. Following this acclimation period, the fish reached an average body weight of approximately 100 g before the initiation of the in vivo gavage trials. During the acclimation period, the water temperature was maintained at 16 ± 2 °C, and dissolved oxygen was kept at 7.4 ± 0.1 mg/L. High-purity fatty acid standards, including oleic acid (OA, purity ≥99% (HPLC), Cat No. O108485), linoleic acid (LA, analytical standard, purity ≥99% (GC), Cat No. L100446), and α-linolenic acid (LNA, analytical standard, purity ≥99%, Cat No. L105577), were purchased from Aladdin (Shanghai, China).

### 2.1 Experimental design and treatments

Following acclimation, fish were fasted for 2 days and randomly assigned to 9 experimental tanks using a computer-generated random number sequence to ensure unbiased allocation. The experimental groups were designed as follows:

1. Control group: Distilled water (W).
2. Vehicle control group: Carboxymethyl cellulose sodium (CMC; solvent).
3. Inhibitor group: Zileuton (Z).
4. Fatty acid groups: Oleic acid (OA), Linoleic acid (LA), and Linolenic acid (LNA)
5. Combination groups: Oleic acid + Inhibitor (OA + Z), Linoleic acid + Inhibitor (LA + Z), and Linolenic acid + Inhibitor (LNA + Z).

### 2.2 Oral gavage procedure

All experimental groups were subjected to intragastric administration (oral gavage) once daily for 3 consecutive days, during which time the fish were fasted to ensure complete gastric emptying and eliminate metabolic interference from complex dietary lipids. The specific dosage regimens were as follows: the carboxymethyl cellulose sodium (vehicle control) group and the zileuton (inhibitor) group received a daily dosage of 100 mg/kg; each fatty acid group received a daily dosage of 400 mg/kg; and the fatty acid + inhibitor combination groups received a daily dosage of 400 mg/kg of the respective fatty acid plus 100 mg/kg of zileuton. The dosages were selected as acute experimental exposure levels to compare short-term responses to different plant-derived C18 fatty acids and to pharmacological inhibition of 5-LOX under controlled conditions. The 400 mg/kg fatty acid dose was used as a standardized acute exposure level for treatment comparison in the present gavage model, rather than to reproduce commercial feeding conditions. The 100 mg/kg Zileuton dose was adapted from acute vertebrate models as an exploratory pharmacological condition, with its safety in teleosts supported by prior research (Costa et al., 2022). Both dosages were empirically confirmed to be effective and non-toxic in preliminary trials. In the present study, zileuton-associated effects were evaluated indirectly through changes in detectable 5-LOX protein abundance and volatile profiles; direct enzymatic activity or target engagement was not measured. In addition, no significant difference was observed between the 0.5% CMC-Na vehicle group and the distilled water control in detectable 5-LOX protein abundance or volatile profiles under the conditions tested.

The oral gavage procedure was established based on previous studies (Collymore et al., 2013; Dang et al., 2016) and optimized through preliminary experiments. A 2-mL syringe fitted with a soft medical silicone infusion hose was used as the gavage apparatus to prevent esophageal mucosal injury. Following MS-222 anesthesia, fish were wrapped in a moist towel to immobilize them and maintain skin integrity. The infusion hose was gently inserted through the oral cavity into the esophagus to a depth of approximately 2–3 cm (determined by preliminary trials). Upon observing swallowing reflexes and confirming smooth insertion without resistance, the liquid was administered smoothly and swiftly. To minimize hypoxic stress, the entire handling process was conducted rapidly, strictly limiting total air exposure to less than 30 s per fish. After gavage, the fish was held in a vertical position with the head upward (partially submerged in the recovery water) for approximately 30 s to prevent regurgitation. The fish were then returned to their respective tanks and closely monitored until the full recovery of normal swimming behavior.

No post-procedural analgesics were administered in this study. Fish were handled rapidly during gavage and monitored after the procedure until normal swimming behavior resumed.

### 2.3 Reagents and preparation

Anesthetic (MS-222; Sigma-Aldrich, St. Louis, MO, USA): An optimal concentration was determined via preliminary tests according to the manufacturer's instructions, defined as the dosage where fish exhibited slow swimming when touched and recovered active swimming within 3 min of being returned to fresh water.

0.5% CMC-Na solution: Precisely weighed 2 g of carboxymethyl cellulose sodium powder (sigma-Aldrich) was dissolved in 400 mL of distilled water

Zileuton Suspension: Accurately weighed zileuton (MedChemExpress, Monmouth Junction, NJ, USA) —a pharmacological inhibitor widely used in studies of 5-LOX-related pathways— was suspended in the 0.5% CMC-Na solution to achieve a final concentration of 5 mg/mL.

### 2.4 Sample collection

At the end of the gavage period, three fish were randomly selected from each tank and anesthetized with MS-222 (30–40 g/m^3^). Muscle samples were rapidly dissected on ice, placed in cryotubes, snap-frozen in liquid nitrogen, and stored at −80 °C for subsequent analysis.

### 2.5 RNA extraction and real-time qPCR analysis

Total RNA was extracted from tissues using a total RNA extraction kit (Thermo Fisher Scientific, Waltham, MA, USA). The concentration and purity of the extracted RNA were quantified using a NanoDrop 2000 spectrophotometer (Thermo Fisher Scientific, Waltham, MA, USA), ensuring that all samples met the quality threshold of an A260/A280 ratio between 1.8 and 2.1. Subsequently, 1 μg of total RNA was utilized for first-strand cDNA synthesis via the PrimeScript RT reagent Kit with gDNA Eraser (Perfect Real Time) (TaKaRa Bio, Kusatsu, Japan), following the protocol for genomic DNA elimination and reverse transcription.

Real-time PCR primers targeting the rainbow trout 5-LOX CDS (NCBI database) were designed via Primer Premier 5.0 (PREMIER Biosoft International, Palo Alto, CA, USA); specific sequences are detailed in Table 1. The qPCR assays were executed using TB Green Premix Ex Taq II (Tli RNaseH Plus) (TaKaRa Bio, Kusatsu, Japan) on a LightCycler 480 II instrument (Roche Diagnostics, Basel, Switzerland). Each 20 μL reaction system contained 10 μL of TB Green Premix, 0.8 μL of each specific primer (10 μM), 2 μL of cDNA template, and 6.4 μL of ddH_2_O. The amplification protocol comprised an initial denaturation at 95 °C for 30 s, followed by 40 cycles of 95 °C for 5 s and 60 °C for 30 s. A melting curve analysis (60–95 °C) was performed post-amplification to verify reaction specificity. *β-actin* served as the internal reference, and relative expression levels were calculated via the 2^-ΔΔCt^ method.

**Table 1.** Primers for the analysis of 5-lipoxygenase gene expression in triploid rainbow trout.

Target gene	Gene Name	Primer sequence (5′-3′)	Genbank accession No.	Amplification Efficiency (%)	*R*^*2*^
β-Actin	Beta actin	F: TACAACGAGCTGAGGGTGGC R: GGCAGGGGTGTTGAAGGTCT	AJ438158.1	101.2	> 0.99
5-LOX	Arachidonate 5-lipoxygenase	F: TCGTCATCTTTACTGCCTCTGC R: CCACCTCGCCTTTCTTCATAG	NM_001303262.1	98.4	> 0.99

Notes: 1). F, forward primer; R, reverse primer; R^2^, coefficient of determination.

2). β-actin was used as the internal reference gene for normalisation.

3). All GenBank accession numbers are from the NCBI database.

### 2.6 5-Lipoxygenase (5-LOX) protein abundance detection

To assess detectable 5-LOX protein abundance across different tissues, tissue samples (12 tissues including gills and liver from the market-size ∼3.0 kg cohort) were homogenized in pre-chilled physiological saline to obtain a strictly standardized 5% (w/v) homogenate (0.05 g tissue per 0.95 mL buffer). The homogenates were centrifuged at 4000 rpm for 10 min at 4 °C using a refrigerated centrifuge (Eppendorf, Hamburg, Germany), and the supernatants were collected. Detectable 5-LOX protein abundance was measured using a Fish-specific Arachidonate 5-Lipoxygenase (ALOX-5) ELISA Kit (MyBioSource, San Diego, CA, USA), following the manufacturer's instructions. Given the high sequence homology of 5-LOX among teleosts, this fish-specific competitive ELISA was employed. In the present study, ELISA-derived values were interpreted as estimates of detectable 5-LOX-related protein abundance rather than direct measurements of enzymatic activity. In addition, although ELISA-derived abundance patterns were broadly compared with qPCR-based transcript trends, such consistency was not regarded as formal analytical validation of antibody specificity in rainbow trout tissues. Briefly, to ensure the spectrophotometric readings fell within the optimal linear detection range of the standard curve (R^2^ > 0.99), the tissue supernatants were diluted 10-fold prior to the assay. Fifty microliters of the diluted samples or standards and 100 μL of HRP-labelled anti-5-LOX antibody were added to the microplate wells. The plate was sealed and incubated at 37 °C for 60 min. After washing five times with wash buffer, 50 μL each of Substrate A and B were added. Following incubation at 37 °C in the dark for 15 min, the reaction was stopped, and absorbance was measured at 450 nm using a microplate reader (Thermo Fisher Scientific, Waltham, MA, USA). The ELISA readout was converted using the corresponding dilution factor and homogenate mass-to-volume ratio. The final detectable 5-LOX protein abundance was standardized to the tissue wet weight and expressed as ng/g tissue.

### 2.7 Analysis of volatile compounds

Volatile compounds were identified and quantified using methods described previously (Ma et al., 2020) with specific instrumental parameters detailed below to ensure analytical reproducibility. Briefly, 3 g of minced muscle sample from each fish and 4.5 mL of saturated NaCl solution were added to a 15-mL headspace vial. The mixture was homogenized on ice using an electric homogenizer (XHF-D, Ningbo Scientz Biotechnology Co., Ltd., Ningbo, China). After adding 15 μL of 2,4,6-trimethylpyridine (91.7 ng/μL; Sigma-Aldrich, St. Louis, MO, USA) as an internal standard (I.S.), the vial was sealed with a screw cap fitted with a septum.

Volatile compounds were extracted using automated solid-phase microextraction (SPME; AOC-6000, CTC Analytics AG, Zwingen, Switzerland) equipped with a 50 μm polydimethylsiloxane (PDMS) and 30 μm divinylbenzene (DVB) fiber (Supelco, Bellefonte, PA, USA). The samples were equilibrated at 60 °C for 20 min, followed by extraction at the same temperature for 35 min.

The extracted analytes were analyzed by gas chromatography–mass spectrometry (GC–MS; QP2020, Shimadzu Corp., Kyoto, Japan). Separation was achieved using an Rtx-5 MS capillary column (30 m × 0.25 mm, 0.25 μm film thickness; Restek Corporation, Bellefonte, PA, USA). Helium was used as the carrier gas at a constant flow rate of 1.5 mL/min. The injector was maintained at 250 °C in splitless mode. The optimized oven temperature program was: initial temperature at 35 °C (held for 3 min), increased to 200 °C at a rate of 10 °C/min, and finally increased to 260 °C at a rate of 5 °C/min (held for 8 min). The mass spectrometer was operated in electron impact (EI) mode (70 eV). The temperatures for the ion source, interface, and quadrupole were set at 230 °C, 280 °C, and 150 °C, respectively, with a full-scan mass range of 30–500 *m*/*z*. Identification was based on linear retention indices (calculated using a series of n-alkanes) and comparison with authentic chemical standards (Sigma-Aldrich, St. Louis, MO, USA).

Quantification was performed using the ratio of the peak area of the analyte to that of the internal standard. The estimated concentration of each volatile compound was calculated as follows:

$$\text{Estimated Concentration}\;\left(\mathrm{ng}/g\right)=\left(\text{Peak Area}\_\text{volatile}/\text{Peak Area}\_I.S.\right)\times \left(\text{Mass of}\;I.S./\text{Mass of sample}\right)$$

Odor activity values (OAVs) were used as comparative indicators of the potential odor relevance of individual volatile compounds under the present analytical framework. Compounds with an OAV ≥ 1 were considered potentially odor-relevant under the adopted criterion, rather than direct evidence of sensory impact in the trout muscle matrix. The OAV was calculated as:

OAV=Ci/OTi

where Ci is the concentration of the volatile compound, and OTi is its odor threshold value reported in the literature (Ma et al., 2020). Because these threshold values were literature-derived and are commonly based on aqueous or simplified systems rather than the trout muscle matrix itself, the resulting OAVs were interpreted cautiously as comparative estimates rather than direct sensory outcomes. Accordingly, OAVs in the present study were used to support relative comparison among treatment groups, whereas confirmation of actual sensory impact would require dedicated sensory evaluation and/or matrix-specific threshold determination.

### 2.8 Statistical analysis

The sample size (*n* = 3 tank-level biological replicates per group) was determined with reference to comparable lipid metabolism studies in salmonids. Statistical analysis was performed using the tank, rather than the individual fish, as the experimental unit to avoid pseudoreplication. For each treatment group, three replicate tanks were used, and three fish were sampled from each tank and averaged to yield one tank-level biological replicate. To reduce subjective bias, investigators conducting the ELISA, qPCR, and GC–MS analyses were blinded to treatment allocation. Technical replicates were included to improve analytical consistency: RT-qPCR was performed in triplicate, whereas ELISA and SPME-GC–MS measurements were performed in duplicate. All data are presented as mean ± standard error (SE).

Statistical analyses were conducted using SPSS 25.0 software (IBM Corp., Armonk, NY, USA). Given the limited tank-level replication and the number of treatment groups and analytical endpoints, all inferential results were interpreted cautiously. Prior to parametric analysis, normality and homogeneity of variance were assessed using the Shapiro-Wilk test and Levene's test, respectively; however, with such small sample sizes, these tests were used only as routine reference checks and were not regarded as strong confirmation that model assumptions were fully satisfied.

For global comparisons among multiple experimental groups, one-way analysis of variance (ANOVA) was used as the primary multi-group analytical approach. When a significant overall effect was detected, Tukey's honestly significant difference (HSD) post hoc test was applied for multiple comparisons among groups. In addition, a limited number of predefined pairwise comparisons were conducted between each fatty-acid treatment and its corresponding zileuton condition to address the inhibitor-related study question. These comparisons were not intended as unrestricted exploratory testing across all possible group combinations. Statistical significance was defined as *P* < 0.05. All graphs were generated using GraphPad Prism 8 software (GraphPad Software, San Diego, CA, USA).

## 3 Results

### 3.1 Tissue distribution of 5-LOX mRNA expression in triploid rainbow trout

**Fig. 1:**
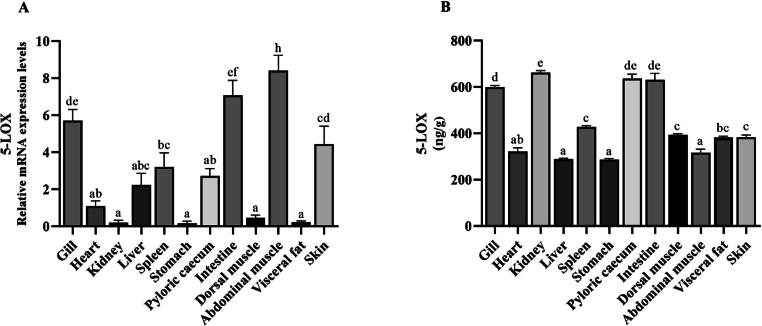
Tissue distribution of 5-lipoxygenase expression in triploid rainbow trout: A) Relative expression levels of 5-LOX mRNA in different tissues. B) Distribution of 5-LOX protein abundance across different tissues. Data are presented as mean ± standard error (mean ± SE, *n* = 3). Different letters above the bar graphs indicate significant differences between groups (*P* < 0.05).

The relative 5-LOX mRNA expression across various tissues is presented in Fig. 1A. A significant tissue effect was observed for 5-LOX mRNA expression (one-way ANOVA, F_11,24_ = 87.58, *P* < 0.001, η^2^ = 0.976), indicating a very large effect size. Transcript abundance was numerically highest in abdominal muscle, followed by the intestine, whereas comparatively low basal expression levels were observed in the kidney, stomach, dorsal muscle, and intraperitoneal fat. Post hoc comparisons showed that abdominal muscle expression was significantly higher than that in most other tissues, while no significant difference was detected between abdominal muscle and intestine. Overall, a tissue-specific distribution pattern of 5-LOX transcription was observed in triploid rainbow trout.

### 3.2 Detectable 5-LOX protein abundance in different tissues

The detectable 5-LOX protein abundance across different tissues is shown in Fig. 1B. Detectable 5-LOX protein abundance differed significantly among tissues (one-way ANOVA, F_11,24_ = 3.80, *P* = 0.003, η^2^ = 0.635), indicating a large tissue effect. The kidney showed the highest detectable 5-LOX protein abundance. The pyloric caeca and intestine also showed relatively high levels, while the gills remained higher than several lower-abundance tissues but lower than the kidney. In contrast, the liver and stomach showed the lowest detectable abundance, whereas the spleen, dorsal muscle, skin, intraperitoneal fat, heart, and abdominal muscle displayed intermediate or relatively low levels. Overall, tissue-related variation in detectable 5-LOX protein abundance was observed.

### 3.3 Effect of different fatty acids on detectable 5-LOX protein abundance

**Fig. 2:**
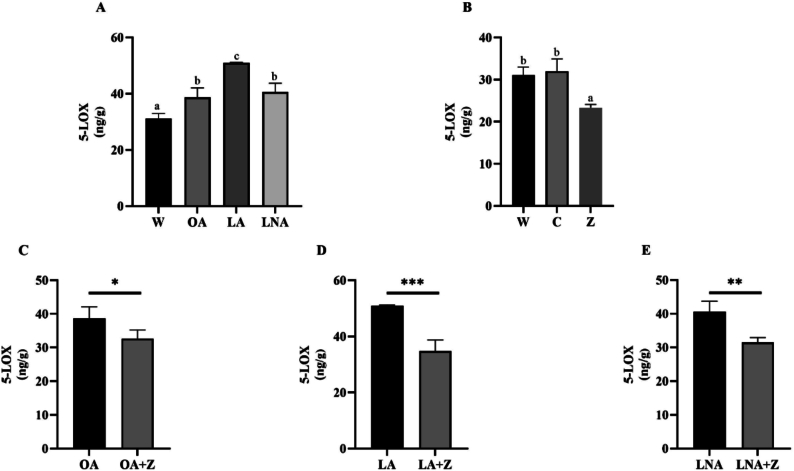
The effects of oral administration of different fatty acids and inhibitors on 5-lipoxygenase protein abundance in abdominal muscle tissue: A) The effects of oleic acid (OA), linoleic acid (LA) and α-linolenic acid (LNA) on 5-LOX protein abundance, compared with a control group administered distilled water (W). B) Changes in 5-LOX protein abundance in abdominal muscle tissue following gavage with distilled water (W) and treatment with the solvent sodium carboxymethylcellulose (C) and the inhibitor zileuton (Z). C–E) Comparative analysis of 5-LOX protein abundance following administration of oleic acid, linoleic acid and linolenic acid (OA, LA, LNA) alone, and in combination with the inhibitor zileuton (OA + Z, LA + Z, LNA + Z). Data are expressed as mean ± standard error (mean ± SE, *n* = 3). Different lowercase letters (a–c) indicate significant differences between groups (*P <* 0.05). Asterisks indicate significant differences between pairs of groups (* denotes *p <* 0.05, ** denotes *p <* 0.01, *** denotes *p <* 0.001).

The effects of acute exposure to different C18 fatty acids on detectable 5-LOX protein abundance in abdominal muscle are shown in Fig. 2A. Detectable 5-LOX protein abundance differed significantly among the W, OA, LA, and LNA groups (one-way ANOVA, F_3,8_ = 37.32, *P* < 0.001, η^2^ = 0.933), indicating a very large treatment effect. Post hoc comparisons showed that the LA group exhibited significantly higher detectable 5-LOX protein abundance than the OA, LNA, and W groups, whereas both the OA and LNA groups were significantly higher than the W group. No significant difference was observed between the OA and LNA groups. These results show that acute fatty acid exposure was associated with increased detectable 5-LOX protein abundance in abdominal muscle, with the largest increase observed in the LA group.

### 3.4 Effect of zileuton on detectable 5-LOX protein abundance

The effect of zileuton treatment on detectable muscle 5-LOX protein abundance is shown in Fig. 2B. A significant group effect was observed among the W, CMC, and Z groups (one-way ANOVA, F_2,6_ = 13.52, *P* = 0.006, η^2^ = 0.818), again indicating a large effect size. Post hoc comparisons showed that the Z group had significantly lower detectable 5-LOX protein abundance than both the CMC vehicle group and the distilled water control group, whereas no significant difference was observed between the CMC vehicle group and the distilled water control group. Under the present experimental conditions, zileuton treatment was associated with lower detectable 5-LOX protein abundance, while no measurable difference was observed between the two control groups.

### 3.5 Combined effects of fatty acids and zileuton on detectable 5-LOX protein abundance

The combined effects of fatty acid treatment and zileuton on detectable 5-LOX protein abundance are presented in Fig. 2C–E. In the OA model, detectable 5-LOX protein abundance was lower in the OA + Z group than in the OA group; however, this reduction did not reach statistical significance in the current dataset (t_4_ = 2.47, *P* = 0.069, η^2^ = 0.604), although the effect size remained substantial. In contrast, zileuton significantly decreased detectable 5-LOX protein abundance in both the LA + Z group (Welch's *t* = 7.13, df = 2.02, *P* = 0.019, η^2^ = 0.962) and the LNA + Z group (Welch's *t* = 4.59, df = 2.80, *P* = 0.022, η^2^ = 0.882) relative to their corresponding fatty acid-only groups. Taken together, lower detectable 5-LOX protein abundance was observed in the inhibitor-treated groups, with statistically significant differences in the LA and LNA models and a similar directional pattern in the OA model.

### 3.6 Effects of fatty acid and inhibitor treatments on volatile compounds in muscle

#### 3.6.1 Effects of individual fatty acids on volatile profiles

The effects of different fatty acids on muscle volatile organic compounds (VOCs) are summarized in Table 2. The total odor-active compound level differed among treatments. Specifically, the TOAC values in the OA group (495.7 ± 0.7 ng/g) and LA group (476.1 ± 9.8 ng/g) were significantly higher than those in the distilled water control group (239.8 ± 7.4 ng/g) and the LNA group (223.4 ± 3.0 ng/g), whereas no significant difference was observed between the OA and LA groups or between the control and LNA groups. At the group level, OA and LA showed higher TOAC values than the control and LNA groups.

**Table 2.** Odor characteristics, concentrations of odor-active compounds and odor activity values in the abdominal muscle tissue of triploid rainbow trout treated with oral administration of different fatty acids.

Odor-active compounds	Odor description	Concentrations ng/g (OAV)^1^			
		W	OA	LA	LNA
1-Heptanol	Green, fermented, nutty ^A^	18.0 ± 1.1	24.1 ± 1.2	17.0 ± 0.9	14.7 ± 1.0
		(3.3) ^a^	(4.5) ^b^	(3.2) ^a^	(2.7) ^a^
1-Octen-3-ol	Earthly, mushroom, fermented ^B, G^	62.4 ± 7.1	88.0 ± 6.9	63.7 ± 4.8	47.7 ± 2.9
		(41.6) ^ab^	(58.7) ^b^	(42.4) ^ab^	(31.8) ^a^
2,3-Pentanedione	Buttery, caramel, fruity ^A^	N.D.	N.D.	7.4 ± 0.8	N.D.
		(N.D.)	(N.D.)	(0.3)	(N.D.)
3,5-Octadien-2-one	Green, floral, cucumber ^H^	63.3 ± 6.0	N.D.	68.9 ± 6.1	28.9 ± 2.7
		(0.4)	(N.D.)	(0.5)	(0.2)
Hexanal	Garlic, green, grassy, pungent, fatty, fishy ^A^	15.6 ± 0.3	221.2 ± 54.5	87.0 ± 5.2	34.1 ± 3.0
		(3.5) ^a^	(49.2) ^b^	(19.3) ^a^	(7.6) ^a^
(Z)-4-Heptenal	Fishy, boiled potato ^B^	N.D.	N.D.	N.D.	3.6 ± 1.0
		(N.D.)	(N.D.)	(N.D.)	(0.9)
Octanal	Sweet, orange, floral, pungent, green, fatty ^A^	14.8 ± 1.4 ^a^	38.0 ± 15.1	51.8 ± 6.1	28.6 ± 1.0
		(21.1) ^a^	(39.9) ^a^	(81.5) ^b^	(40.9) ^a^
(E,E)-2,4-Heptadienal	Fishy, grassy ^E^	6.4 ± 0.7	7.5 ± 0.8	N.D.	4.1 ± 0.6
		(0.4)	(0.5)	(N.D.)	(0.3)
(E)-2-Octenal	Moldy, pungent, cucumber, fatty, mushroom ^C, F^	6.0 ± 0.3	4.0 ± 0.9	5.0 ± 0.4	1.6 ± 0.3
		(2.0) ^b^	(1.3) ^b^	(1.7) ^b^	(0.5) ^a^
Nonanal	Geranium, fishy, plastic, orange, green, fatty ^A^	32.2 ± 3.5	75.5 ± 1.9	109.5 ± 5.9	53.6 ± 1.9
		(29.2) ^a^	(68.6) ^c^	(99.5) ^d^	(48.8) ^b^
(E,Z)-2,6-Nonadienal	Cucumber, floral ^G^	5.6 ± 0.6	5.4 ± 1.7	6.6 ± 0.5	3.0 ± 0.4
		(7.0) ^ab^	(6.7) ^ab^	(8.2) ^b^	(3.8) ^a^
(E)-2-Nonenal	Moss, woody, floral, green, fruity ^C, F^	5.2 ± 0.7	N.D.	5.3 ± 0.2	1.9 ± 0.4
		(65.3)	(N.D.)	(65.6)	(28.4)
Decanal	Marine, cucumber, floral, fatty, orange, green ^A^	3.0 ± 0.4	4.6 ± 0.7	10.7 ± 0.9	N.D.
		(1.5)	(2.3)	(5.4)	(N.D.)
(E)-2-Decenal	Orange, fatty ^D^	N.D.	2.8 ± 0.6	2.5 ± 0.2	1.3 ± 0.2
		(N.D.)	(9.4)	(8.3)	(4.2)
Undecanal	Green, aniseed, fruity, minty ^A, F^	N.D.	N.D.	2.9 ± 0.5	1.8 ± 0.3
		(N.D.)	(N.D.)	(0.8)	(0.4)
Total (TOAC)		239.8 ± 7.4 ^a^	495.7 ± 0.7 ^b^	476.1 ± 9.8 ^b^	223.4 ± 3.0 ^a^

W: distilled water; OA: oleic acid; LA: linoleic acid; LNA: linolenic acid. Values are presented as means ± S.E.M. (n = 3 per group) in the same row with different superscript lowercase letters are significantly different (*p* < 0.05). N.D.: not detected.

^A–H^ Odor description was obtained from A: Mu et al. (2017), B: Selli and Cayhan (2009), C: Wu et al. (2014), D: Turchini et al. (2007), E: Fu et al. (2009), F: Varlet et al. (2006), G: Frank et al. (2009), H: Zhou et al. (2016), respectively.

1 Odor-active compounds are volatile compounds with the odor activity values (OAVs, the ratio of concentration and odor threshold of volatile compound) equal to or greater than 1.

In the OA group, the main increases in measured volatile abundance were observed for 1-heptanol, 1-octen-3-ol, hexanal, and nonanal relative to the control group. For these compounds, the corresponding OAVs remained above 1 under the adopted criterion. Among them, hexanal and nonanal showed larger increases than the others. In contrast, (E)-2-nonenal and 3,5-octadien-2-one were not detected in this group.

In the LA group, the main increases in measured volatile abundance were observed for octanal and nonanal relative to the control group, and both compounds showed OAVs above 1. TOAC also increased significantly in this group. Although 2,3-pentanedione and undecanal were detected after LA treatment, their OAVs remained below 1 under the adopted criterion. In addition, (*E,E*)-2,4-heptadienal was not detected in the LA group.

In the LNA group, fewer compounds showed changes in measured volatile abundance than in the OA and LA groups. Nonanal remained higher than in the control group and maintained an OAV above 1. The concentrations of (Z)-4-heptenal and undecanal also increased, although their OAVs remained below 1. Decanal was not detected in this group. Overall, the LNA group showed fewer compound-level changes and a lower TOAC than the OA and LA groups.

#### 3.6.2 Effects of oleic acid and zileuton on volatile compounds

The volatile changes associated with the OA + Z treatment are summarized in Table 3. Compared with the OA group, the OA + Z group showed a lower TOAC, decreasing from 495.7 ± 0.7 to 145.4 ± 1.1 ng/g. This reduction in TOAC represented the main group-level difference between the OA and OA + Z treatments.

**Table 3.** Odor characteristics, concentrations of odor-active compounds and odor activity values in the abdominal muscle tissue of triploid rainbow trout treated with oral administration of oleic acid and oleic acid plus an inhibitor.

Odor-active compounds	Odor description	Concentrations ng/g (OAV)^1^		
		OA	OA + Z	*t*-test
1-Heptanol	Green, fermented, nutty ^A^	24.1 ± 1.2	6.5 ± 0.5	***
		(4.5)	(1.2)	
1-Octen-3-ol	Earthly, mushroom, fermented ^B, G^	88.0 ± 6.9	19.8 ± 1.0	***
		(58.7)	(13.3)	
Hexanal	Garlic, green, grassy, pungent, fatty, fishy ^A^	221.2 ± 54.5	31.6 ± 0.6	***
		(49.2)	(7.0)	
Octanal	Sweet, orange, floral, pungent, green, fatty ^A^	38.0 ± 15.1	14.1 ± 0.0	**
		(39.9)	(20.2)	
(E,E)-2,4-Heptadienal	Fishy, grassy ^E^	7.5 ± 0.8	2.7 ± 0.2	***
		(0.5)	(0.2)	
(E)-2-Octenal	Moldy, pungent, cucumber, fatty, mushroom ^C, F^	4.0 ± 0.9	1.8 ± 0.3	*
		(1.3)	(0.6)	
Nonanal	Geranium, fishy, plastic, orange, green, fatty ^A^	75.5 ± 1.9	38.6 ± 2.1	***
		(68.6)	(35.1)	
(E,Z)-2,6-Nonadienal	Cucumber, floral ^G^	5.4 ± 1.7	2.9 ± 0.2	ns
		(6.7)	(3.6)	
Decanal	Marine, cucumber, floral, fatty, orange, green ^A^	4.6 ± 0.7	5.6 ± 0.5	ns
		(2.3)	(2.8)	
(E)-2-Decenal	Orange, fatty ^D^	2.8 ± 0.6	1.9 ± 0.5	ns
		(9.4)	(6.4)	
Total (TOAC)		495.7 ± 0.7	145.4 ± 1.1	***

OA: oleic acid; OA + Z: oleic acid + Zileuton. Values are presented as means ± S.E.M. (*n* = 3 per group) * denotes *p* < 0.05, ** denotes *p* < 0.01, *** denotes *p* < 0.001.

^A–H^ Odor description was obtained from A: Mu et al. (2017), B: Selli and Cayhan (2009), C: Wu et al. (2014), D: Turchini et al. (2007), E: Fu et al. (2009), F: Varlet et al. (2006), G: Frank et al. (2009), H: Zhou et al. (2016), respectively.

1 Odor-active compounds are volatile compounds with the odor activity values (OAVs, the ratio of concentration and odor threshold of volatile compound) equal to or greater than 1.

At the individual compound level, octanal, nonanal, hexanal, 1-octen-3-ol, (E)-2-octenal, 1-heptanol, and (*E,E*)-2,4-heptadienal were all significantly lower in the OA + Z group than in the OA group. The corresponding OAVs were also reduced, although several compounds remained above 1 where applicable. By contrast, (E,Z)-2,6-nonadienal, decanal, and (E)-2-decenal did not differ significantly between the two groups. Overall, decreases were observed in both TOAC and several individual VOCs in the OA + Z group relative to the OA group.

#### 3.6.3 Effects of linoleic acid and zileuton on volatile compounds

The comparative effects of LA and LA + Z treatments on VOCs are shown in Table 4. Relative to the LA group, the LA + Z group showed a lower TOAC, from 476.1 ± 9.8 to 342.8 ± 3.4 ng/g. This reduction in TOAC represented the main group-level difference between the LA and LA + Z treatments.

**Table 4.** Odor characteristics, concentrations of odor-active compounds and odor activity values in the abdominal muscle tissue of triploid rainbow trout treated with oral administration of linoleic acid and linoleic acid plus an inhibitor.

Odor-active compounds	Odor description	Concentrations ng/g (OAV)^1^		
		LA	LA + Z	T-test
1-Heptanol	Green, fermented, nutty ^A^	17.0 ± 0.9	11.5 ± 0.3	***
		(3.2)	(2.2)	
1-Octen-3-ol	Earthly, mushroom, fermented ^B, G^	63.7 ± 4.8	41.3 ± 1.1	**
		(42.4)	(27.5)	
2,3-Pentanedione	Buttery, caramel, fruity ^A^	7.4 ± 0.8	N.D.	
		(0.3)	(N.D.)	
3,5-Octadien-2-one	Green, floral, cucumber ^H^	68.9 ± 6.1	81.5 ± 1.9	ns
		(0.5)	(0.5)	
Hexanal	Garlic, green, grassy, pungent, fatty, fishy ^A^	87.0 ± 5.2	57.4 ± 1.0	***
		(19.3)	(12.8)	
Octanal	Sweet, orange, floral, pungent, green, fatty ^A^	51.8 ± 6.1	20.9 ± 0.5	***
		(81.5)	(29.9)	
(E)-2-Octenal	Moldy, pungent, cucumber, fatty, mushroom ^C, F^	5.0 ± 0.4	4.3 ± 0.3	*
		(1.7)	(1.4)	
Nonanal	Geranium, fishy, plastic, orange, green, fatty ^A^	109.5 ± 5.9	71.9 ± 5.8	**
		(99.5)	(65.3)	
(E,Z)-2,6-Nonadienal	Cucumber, floral ^G^	6.6 ± 0.5	7.3 ± 0.2	ns
		(8.2)	(9.1)	
(E)-2-Nonenal	Moss, woody, floral, green, fruity ^C, F^	5.3 ± 0.2	4.8 ± 0.6	ns
		(65.6)	(59.9)	
Decanal	Marine, cucumber, floral, fatty, orange, green ^A^	10.7 ± 0.9	9.5 ± 0.4	ns
		(5.4)	(4.7)	
(E)-2-Decenal	Orange, fatty ^D^	2.5 ± 0.2	3.4 ± 0.2	**
		(8.3)	(11.3)	
Undecanal	Green, aniseed, fruity, minty ^A, F^	2.9 ± 0.5	N.D.	
		(0.8)	(N.D.)	
Total (TOAC)		476.1 ± 9.8	342.8 ± 3.4	***

LA: linoleic acid; LA + Z: linoleic acid + Zileuton. Values are presented as means ± S.E.M. (*n* = 3 per group). * denotes *p* < 0.05, ** denotes *p* < 0.01, *** denotes *p* < 0.001.

^A–H^ Odor description was obtained from A: Mu et al. (2017), B: Selli and Cayhan (2009), C: Wu et al. (2014), D: Turchini et al. (2007), E: Fu et al. (2009), F: Varlet et al. (2006), G: Frank et al. (2009), H: Zhou et al. (2016), respectively.

1 Odor-active compounds are volatile compounds with the odor activity values (OAVs, the ratio of concentration and odor threshold of volatile compound) equal to or greater than 1.

More specifically, octanal, nonanal, hexanal, 1-octen-3-ol, 1-heptanol, and (E)-2-octenal were all significantly reduced after zileuton treatment, and the corresponding OAVs also declined. In contrast, (E)-2-decenal showed a significantly higher concentration in the LA + Z group, and its OAV remained above 1 under the adopted criterion. In addition, 2,3-pentanedione and undecanal were not detected in the LA + Z group. Overall, the LA + Z group showed a lower TOAC than the LA group, although the direction of change was not the same for all compounds.

#### 3.6.4 Effects of linolenic acid and zileuton on volatile compounds

The volatile profiles of the LNA and LNA + Z groups are compared in Table 5. Compared with the LNA group, the LNA + Z group showed a lower TOAC, decreasing from 223.4 ± 3.0 to 190.0 ± 2.9 ng/g. This reduction was smaller than those observed in the OA and LA models.

**Table 5.** Odor characteristics, concentrations of odor-active compounds and odor activity values in the abdominal muscle tissue of triploid rainbow trout treated with oral administration of linolenic acid and linolenic acid plus an inhibitor.

Odor-active compounds	Odor description	Concentrations ng/g (OAV)^1^		
		LNA	LNA + Z	T-test
1-Heptanol	Green, fermented, nutty ^A^	14.7 ± 1.0	5.8 ± 0.9	***
		(2.7)	(1.1)	
1-Octen-3-ol	Earthly, mushroom, fermented ^B, G^	47.7 ± 2.9	25.4 ± 1.3	***
		(31.8)	(16.9)	
3,5-Octadien-2-one	Green, floral, cucumber ^H^	28.9 ± 2.7	35.2 ± 1.3	*
		(0.2)	(0.2)	
Hexanal	Garlic, green, grassy, pungent, fatty, fishy ^A^	34.1 ± 3.0	45.6 ± 0.9	**
		(7.6)	(10.1)	
(Z)-4-Heptenal	Fishy, boiled potato ^B^	3.6 ± 1.0	N.D.	
		(0.9)	(N.D.)	
Octanal	Sweet, orange, floral, pungent, green, fatty ^A^	28.6 ± 1.0	18.1 ± 0.4	***
		(40.9)	(25.9)	
(E,E)-2,4-Heptadienal	Fishy, grassy ^E^	4.1 ± 0.6	2.8 ± 0.4	*
		(0.3)	(0.2)	
(E)-2-Octenal	Moldy, pungent, cucumber, fatty, mushroom ^C, F^	1.6 ± 0.3	1.9 ± 0.4	ns
		(0.5)	(0.6)	
Nonanal	Geranium, fishy, plastic, orange, green, fatty ^A^	53.6 ± 1.9	48.8 ± 0.3	*
		(48.8)	(44.3)	
(E,Z)-2,6-Nonadienal	Cucumber, floral ^G^	3.0 ± 0.4	3.5 ± 0.2	ns
		(3.8)	(4.4)	
(E)-2-Nonenal	Moss, woody, floral, green, fruity ^C, F^	1.9 ± 0.4	2.2 ± 0.5	ns
		(28.4)	(27.5)	
(E)-2-Decenal	Orange, fatty ^D^	1.3 ± 0.2	1.4 ± 0.2	ns
		(4.2)	(4.7)	
Undecanal	Green, aniseed, fruity, minty ^A, F^	1.8 ± 0.3	N.D.	
		(0.4)	(N.D.)	
Total (TOAC)		223.4 ± 3.0	190.0 ± 2.9	***

LNA: linolenic acid; LNA + Z: linolenic acid + Zileuton. Values are presented as means ± S.E.M. (n = 3 per group).* denotes *p* < 0.05, ** denotes *p* < 0.01, *** denotes *p* < 0.001.

^A–H^ Odor description was obtained from A: Mu et al. (2017), B: Selli and Cayhan (2009), C: Wu et al. (2014), D: Turchini et al. (2007), E: Fu et al. (2009), F: Varlet et al. (2006), G: Frank et al. (2009), H: Zhou et al. (2016), respectively.

1 Odor-active compounds are volatile compounds with the odor activity values (OAVs, the ratio of concentration and odor threshold of volatile compound) equal to or greater than 1.

At the compound level, octanal, nonanal, 1-octen-3-ol, 1-heptanol, and (*E,E*)-2,4-heptadienal were significantly lower in the LNA + Z group than in the LNA group, and their corresponding OAVs also decreased. In contrast, hexanal and 3,5-octadien-2-one were significantly increased after zileuton treatment. In addition, (Z)-4-heptenal and undecanal were not detected or had OAVs below 1 in the LNA + Z group. Overall, the TOAC value was lower in the LNA + Z group, whereas compound-level changes were mixed.

## 4 Discussion

The increasing use of plant-derived oils in aquafeeds has raised interest in how altered muscle fatty acid composition may affect volatile profiles and, consequently, fillet aroma. Previous studies have shown that replacement of fish oil with plant oils can modify volatile composition in aquatic foods (Turchini et al., 2007; Wu et al., 2014; Xu et al., 2022; Zhou et al., 2016). However, the biochemical processes associated with these changes remain incompletely understood in vivo. In this context, the present study examined tissue distribution of 5-LOX-related expression, acute fatty-acid-associated changes in detectable 5-LOX protein abundance in abdominal muscle, and the accompanying volatile profiles under fatty acid and zileuton treatments in triploid rainbow trout.

LOX-related processes have long been discussed in relation to lipid oxidation and volatile formation, but the specific contribution of individual LOX isoforms in edible fish muscle remains unclear, especially under in vivo conditions. In the present study, 5-LOX was examined as a candidate enzyme potentially associated with volatile changes under acute fatty acid exposure. Using a short-term oral gavage model and zileuton as an exploratory pharmacological tool, the present work aimed to determine whether acute exposure to OA, LA, or LNA would be accompanied by changes in detectable muscle 5-LOX protein abundance and volatile profiles. Within the limits of this design, the results support treatment-associated and inhibitor-sensitive associations between detectable 5-LOX protein abundance and volatile outcomes, but they should not be interpreted as direct proof of enzymatic activity, pathway-specific flux, or causal regulation of volatile formation.

### 4.1 Tissue distribution of 5-LOX expression in triploid rainbow trout

One notable result of this study was that 5-LOX-related signals were not uniformly distributed among tissues. Abdominal muscle showed the highest mRNA expression, whereas kidney exhibited the highest detectable protein abundance, with relatively high detectable protein abundance also observed in pyloric caeca and intestine. This pattern differs from the tissue distribution more commonly associated with the immune-related functions of 5-LOX in vertebrates, where kidney and gill are often discussed as relevant tissues in teleost fish (Cheng et al., 2025; Tharuka et al., 2019).

The lack of direct correspondence between transcript abundance and detectable protein abundance may indicate tissue-specific regulation of 5-LOX-related expression. Such divergence could reflect differences in post-transcriptional regulation, translation efficiency, protein stability, or tissue-specific turnover, although these possibilities were not directly examined in the present study. In abdominal muscle, high transcript abundance together with relatively low basal detectable protein abundance suggests that 5-LOX-related protein signal was present at a low baseline level under unstimulated conditions. This pattern identifies abdominal muscle as a relevant tissue for examining treatment-associated responses, but it does not establish a constitutively active 5-LOX pathway in muscle.

The physiological basis of this tissue pattern remains uncertain. At most, the present data suggest that abdominal muscle may retain the capacity to show treatment-associated 5-LOX-related responses despite relatively low basal detectable protein abundance. However, the underlying regulatory basis was not examined in this study. The present data also do not support strong conclusions regarding triploidy-specific regulation. Because the study used only an all-female triploid model and did not include diploid controls, the observed expression pattern cannot be attributed specifically to triploidy. Therefore, triploid rainbow trout should be regarded here primarily as the experimental model used rather than as the basis for a distinct mechanistic interpretation.

### 4.2 Fatty acid exposure, detectable 5-LOX protein abundance, and volatile responses

A central finding of this study was that acute exposure to different fatty acids was associated with distinct changes in detectable 5-LOX protein abundance in muscle. Among the tested fatty acids, LA produced the largest increase in detectable protein abundance, whereas OA and LNA also increased the detectable signal relative to the water control group. These results indicate that short-term fatty acid exposure was associated with altered detectable 5-LOX protein abundance under the present experimental conditions.

The stronger responses observed in the LA and LNA groups are consistent with the structural characteristics of polyunsaturated fatty acids, particularly the presence of multiple double bonds that are relevant to lipid oxidation processes (Yang et al., 2017; Yao et al., 2024). Even so, the present data do not establish that the observed changes were driven specifically by direct 5-LOX catalysis of the administered substrates. This distinction is important because the assay used here quantified detectable protein abundance rather than enzymatic activity.

The OA-related response requires particularly cautious interpretation. As a monounsaturated fatty acid, OA lacks the classical *cis,cis*-1,4-pentadiene structure usually associated with lipoxygenase oxygenation. Therefore, the concurrent increase in detectable 5-LOX protein abundance and the associated volatile changes following OA treatment should not be interpreted as direct evidence that OA itself was oxidized by 5-LOX. Several non-exclusive explanations remain possible, including trace PUFA contamination in the OA preparation, altered endogenous lipid mobilization, or indirect effects on muscle lipid metabolism and signalling. However, these possibilities were not directly tested in the present study. Accordingly, the OA-associated response is better interpreted as a treatment-related pattern rather than as evidence of direct substrate-specific catalysis.

### 4.3 Zileuton-sensitive associations between detectable 5-LOX protein abundance and volatile profiles

The zileuton treatment results showed that detectable 5-LOX protein abundance decreased relative to control conditions and was also lower in the LA + Z and LNA + Z groups than in the corresponding fatty-acid-only groups. In the OA model, the same directional pattern was observed, although the difference did not reach statistical significance in the current dataset. At the volatile level, zileuton treatment was accompanied by lower TOAC values in all three fatty-acid models, together with reductions in several individual VOCs. These results indicate that the observed protein and volatile patterns were inhibitor sensitive under the present conditions.

This inhibitor-sensitive pattern is consistent with possible involvement of 5-LOX-related processes in the observed volatile changes. However, the strength of inference should not be overstated. The present study did not directly measure 5-LOX enzymatic activity, target engagement, or pathway-specific flux. Therefore, the data do not demonstrate that 5-LOX was the sole or direct causal regulator of the affected volatiles. Instead, the results support an association between zileuton-sensitive changes in detectable 5-LOX protein abundance and alterations in volatile profiles under acute fatty acid exposure.

It is also important to avoid overinterpreting the decrease in detectable protein abundance after zileuton treatment. The present dataset does not allow distinction among reduced protein stability, altered expression, indirect metabolic feedback, or assay-level effects associated with inhibitor treatment. Accordingly, these results should be interpreted conservatively as inhibitor-sensitive changes in detectable protein abundance rather than as direct evidence of altered catalytic function.

### 4.4 Volatile profiles and pathway interpretation

The VOC data showed clear group-level differences in overall volatile profiles. OA and LA treatments were associated with higher TOAC values than the control and LNA groups, whereas the LNA group remained closer to the control at the overall level. At the compound level, OA and LA were associated with broader changes in aldehydes and alcohols, whereas the LNA response involved fewer clear changes. These findings indicate that the three tested fatty acids were associated with distinct volatile patterns under the present acute treatment model.

Zileuton treatment lowered TOAC values in all three fatty-acid models and reduced several individual compounds, although the direction and magnitude of change were not uniform across all VOCs. Some compounds remained unchanged, and a small number increased under inhibitor treatment. This pattern suggests that the volatile response cannot be explained by a single measured factor alone. However, the present study does not provide direct evidence regarding how flux was redistributed among competing oxidative routes.

For this reason, interpretations regarding how the flux was specifically redirected through other unmeasured oxidative pathways should remain speculative. These possibilities may warrant future investigation, but they were not directly measured here and should not be presented as established mechanisms. At present, the most appropriate interpretation is that zileuton-sensitive treatment effects were observed in both detectable 5-LOX protein abundance and volatile profiles, while the contribution of other oxidative processes remains unresolved.

### 4.5 OAV interpretation, model scope, and study limitations

The OAV and TOAC analyses were useful for comparing the relative odor-active potential of treatment groups under the adopted criterion and for prioritising compounds that may warrant further attention. However, these calculations should not be interpreted as direct sensory outcomes. The odor thresholds used to calculate OAVs were derived from literature sources and are not specific to the trout muscle matrix examined in this study. In addition, volatile release in fish muscle is influenced by matrix-dependent factors such as lipid partitioning, protein binding, and other physicochemical interactions (Frank et al., 2009; Selli & Cayhan, 2009; Varlet et al., 2006). Because sensory evaluation was not performed, the present OAV-based discussion should be regarded as a comparative analytical framework rather than direct evidence of flavour perception or consumer-relevant sensory intensity.

Several additional limitations should also be considered. First, the experimental model was based on a 3-day acute oral gavage protocol in juvenile fish and therefore represents a short-term exposure model rather than a chronic feeding strategy. As such, the present findings should not be treated as directly equivalent to commercial dietary fish-oil replacement conditions. Instead, they reflect acute treatment-associated chemical responses under controlled experimental conditions. Second, effective biological replication was limited, which constrains statistical resolution across multiple treatment groups and analytical endpoints. Third, detectable 5-LOX protein abundance was measured, but enzymatic activity was not. Fourth, zileuton-associated effects were evaluated indirectly through protein abundance and volatile changes rather than through direct assays of target engagement or pathway flux. Finally, the OA-associated response remains open to alternative explanations, including possible trace PUFA contamination or indirect metabolic effects, because independent compositional verification and isotope-based tracing were not performed.

Despite these limitations, the present study provides useful evidence that acute exposure to different plant-derived C18 fatty acids was associated with distinct volatile patterns in triploid rainbow trout muscle and that several of these patterns were sensitive to zileuton treatment. Within the boundaries of the current design, these findings indicate inhibitor-sensitive associations between acute fatty acid exposure, detectable 5-LOX protein abundance, and volatile changes, while the underlying biochemical mechanisms remain unresolved. Future studies should therefore combine direct enzyme activity measurements, target-specific pathway analyses, isotope tracing, comparison with other oxidative pathways, sensory evaluation, and longer-term feeding trials to clarify more precisely how fatty acid composition, 5-LOX-related oxidation, and volatile formation are linked in fish muscle.

## 5 Conclusion

In conclusion, the present study showed that acute exposure to plant-derived C18 fatty acids was associated with changes in detectable 5-lipoxygenase (5-LOX) protein abundance and volatile profiles in the muscle of triploid rainbow trout. Linoleic acid showed the clearest increase in detectable 5-LOX protein abundance among the tested fatty acids, and different fatty acid treatments were associated with distinct volatile patterns under the present acute exposure model. Zileuton treatment was also associated with changes in detectable 5-LOX protein abundance and several volatile measures, indicating inhibitor-sensitive associations under the conditions tested. However, because the present study did not directly measure enzymatic activity, target engagement, competing oxidative flux, or sensory perception, these findings should be interpreted cautiously. Overall, the present results provide preliminary evidence that acute plant-derived C18 fatty acid exposure is associated with volatile changes in trout muscle, while the underlying biochemical mechanisms remain unresolved. These findings may help inform future studies on fatty acid composition, oxidative pathways, and volatile formation in fish muscle.

## CRediT authorship contribution statement

**Yongbang Si:** Writing – review & editing, Writing – original draft, Methodology, Investigation, Formal analysis, Data curation. **Yongna Song:** Methodology, Investigation, Formal analysis, Data curation. **Dong Huang:** Methodology, Data curation. **Yifei Chen:** Methodology, Investigation, Data curation. **Jun Sun:** Software, Methodology. **Zezhong Wu:** Methodology, Investigation. **Rui Ma:** Supervision, Resources, Methodology. **Guoliang Sun:** Writing – review & editing, Writing – original draft, Supervision, Resources, Methodology, Formal analysis. **Yuqiong Meng:** Writing – review & editing, Writing – original draft, Supervision, Resources, Methodology.

## Declaration of competing interest

The authors declare that they have no known competing financial interests or personal relationships that could have appeared to influence the work reported in this paper.

## Data Availability

Data will be made available on request.
